# Toward Artificial Intelligence in Oncology and Cardiology: A Narrative Review of Systems, Challenges, and Opportunities

**DOI:** 10.3390/jcm14217555

**Published:** 2025-10-24

**Authors:** Visar Vela, Ali Yasin Sonay, Perparim Limani, Lukas Graf, Besmira Sabani, Diona Gjermeni, Andi Rroku, Arber Zela, Era Gorica, Hector Rodriguez Cetina Biefer, Uljad Berdica, Euxhen Hasanaj, Adisa Trnjanin, Taulant Muka, Omer Dzemali

**Affiliations:** 1 Centre Suisse de Contrôle de Qualité, 1225 Geneva, Switzerland; 2 Epistudia, 3008 Bern, Switzerland; 3 Department of Visceral Surgery and Transplantation, University Hospital Zurich, 8091 Zurich, Switzerland; 4 Center for Laboratory Medicine, Hemostasis and Hemophilia Center, 9008 St. Gallen, Switzerland; 5 Institute of Chemistry and Biotechnology, Zurich University of Applied Sciences (ZHAW), 8820 Wädenswil, Switzerland; 6 Department of Cardiology and Angiology, University Heart Center Freiburg-Bad Krozingen, Faculty of Medicine, University of Freiburg, 79085 Freiburg, Germany; 7 Stanford Center for Clinical Research, Department of Medicine, Stanford University School of Medicine, Stanford, CA 94305, USA; 8 Department of Cardiology, Angiology and Intensive Care Medicine, Deutsches Herzzentrum der Charité, Charité-Universitätsmedizin Berlin (Campus Benjamin Franklin), 12203 Berlin, Germany; 9 DZHK German Center for Cardiovascular Research, Partner Site Berlin, 13353 Berlin, Germany; 10 Department of Computer Science, University of Freiburg, 79085 Freiburg, Germany; 11 Center for Translational and Experimental Cardiology (CTEC), Department of Cardiology, University Hospital Zurich, University of Zürich, Wagistrasse 12, 8952 Schlieren, Switzerland; 12 Department of Cardiac Surgery, City Hospital Zurich-Triemli, 8063 Zurich, Switzerland; 13 Oxford Robotics Institute, Department of Engineering Science, University of Oxford, Oxford 1096, UK; 14 GenBio AI, Palo Alto, CA 94301, USA

**Keywords:** artificial intelligence, machine learning, deep learning, oncology, cardiology, clinical diagnostics

## Abstract

**Background:** Artificial intelligence (AI), the overarching field that includes machine learning (ML) and its subfield deep learning (DL), is rapidly transforming clinical research by enabling the analysis of high-dimensional data and automating the output of diagnostic and prognostic tests. As clinical trials become increasingly complex and costly, ML-based approaches (especially DL for image and signal data) offer promising solutions, although they require new approaches in clinical education. **Objective:** Explore current and emerging AI applications in oncology and cardiology, highlight real-world use cases, and discuss the challenges and future directions for responsible AI adoption. **Methods:** This narrative review summarizes various aspects of AI technology in clinical research, exploring its promise, use cases, and its limitations. The review was based on a literature search in PubMed covering publications from 2019 to 2025. Search terms included “artificial intelligence”, “machine learning”, “deep learning”, “oncology”, “cardiology”, “digital twin”. and “AI-ECG”. Preference was given to studies presenting validated or clinically applicable AI tools, while non-English articles, conference abstracts, and gray literature were excluded. **Results:** AI demonstrates significant potential in improving diagnostic accuracy, facilitating biomarker discovery, and detecting disease at an early stage. In clinical trials, AI improves patient stratification, site selection, and virtual simulations via digital twins. However, there are still challenges in harmonizing data, validating models, cross-disciplinary training, ensuring fairness, explainability, as well as the robustness of gold standards to which AI models are built. **Conclusions:** The integration of AI in clinical research can enhance efficiency, reduce costs, and facilitate clinical research as well as lead the way towards personalized medicine. Realizing this potential requires robust validation frameworks, transparent model interpretability, and collaborative efforts among clinicians, data scientists, and regulators. Interoperable data systems and cross-disciplinary education will be critical to enabling the integration of scalable, ethical, and trustworthy AI into healthcare.

## 1. Introduction

Artificial intelligence (AI) is transforming modern medicine by enabling the use of vast and complex datasets for analysis. Outcomes include uncovering patterns, predicting results, and supporting human healthcare decisions in multiple domains [1,2]. Within AI, machine learning (ML) refers to algorithms that learn patterns from data to make predictions without explicit rule-based programming, and deep learning (DL) is a specialized subset of ML that uses multi-layered neural networks, such as convolutional neural networks (CNNs) to model highly complex relationships in images, signals, and text.

In the medical field, cardiology and oncology are especially suitable for novel AI-driven innovations [1]. Cardiovascular diseases remain the leading cause of death in the world, followed by cancer accounting for 17.9 million and 10 million deaths per year, respectively [3,4]. In oncology, AI is facilitating earlier detection through digital pathology [5], liquid biopsy analysis and sequencing [6], while in cardiology, DL-powered image and signal interpretation is advancing the accuracy of electrocardiograms (ECG) and radiological imaging [1,6,7].

Recent research enhances the explainability of deep learning models in pathology, exemplified by the Vision Transformer with Adaptive Model fusion and multiobjective optimization (ViT-AMC) [8]. This model integrates transformer-based feature extraction and attention mechanisms to emphasize diagnostically significant areas in laryngeal tumor grading from histopathological images [8,9]. Such explainable artificial intelligence (XAI) frameworks are crucial for gaining regulatory acceptance and fostering physician confidence in AI-driven oncology diagnosis [10].

Clinical practice now generates vast data, such as omics [11], imaging [12], electronic health records (EHRs) [13], and wearables. Harnessing these sources with AI can accelerate discovery and diagnostic scaling [14]. These large datasets from both preclinical and clinical research led to the training of foundation models (FMs) such as BioGPT, Med-Pal M and PathGPT that can capture biological and clinical patterns more comprehensively than models trained on small, narrowly defined datasets. Once pretrained, FMs can be fine-tuned for downstream tasks, often showing strong performance even with limited labeled data [15].

Yet, these opportunities present significant challenges as ML models access sensitive patient data. Therefore, it is essential to prioritize privacy, standardization, and interoperability of these models before their clinical translation [1,2]. In parallel, this digital transformation calls for broader systemic changes, including novel regulations and clinical training of both healthcare personnel and data scientists. Big datasets do not automatically yield high-quality clinical evidence; model performance is frequently limited by measurement noise, systematic missingness, batch effects across sites/instruments, label or annotation error, population and sampling bias, and temporal drift in both biology and care pathways [16].

Furthermore, AI plays a crucial role in clinical trials [17] by facilitating patient recruitment, improving trial retention and stratification, simulating outcomes with digital twins [1,2,18], and uncovering new insights from real-world evidence (RWE) following treatment approval [19]. AI can support digital health and software as a medical device approval by enabling virtual and hybrid trials that reduce participant and site burden. The power that AI offers to real-time analysis of complex datasets also improves trial efficiency [1,2].

While most systematic reviews concentrate on a single disease area or a specific AI modality, in this narrative review, we offer a broader perspective by comparing applications across both oncology and cardiology. It also places particular emphasis on the practical challenges of translation, including data interoperability, regulatory approval, and integration into clinical workflows. By focusing on both opportunities and pitfalls, we aim to provide clinicians, data scientists, and policymakers with a roadmap for responsibly and scalably integrating AI in these high-impact fields.

## 2. Methods

This article was designed as a narrative review aimed at synthesizing current knowledge on the application of AI in oncology and cardiology. A comprehensive, non-systematic search of the literature was conducted across major databases, including PubMed covering publications from 2019 to 2025. Relevant studies, reviews, and reports were identified using combinations of the following keywords: “artificial intelligence”, “machine learning”, “deep learning”. “explainable AI”, “large language model”, “oncology”, “cancer”, “cardiology”, “cardiovascular”, “clinical decision support”, “diagnostics”, and “medical imaging”. Additional sources, including white papers and institutional reports, were reviewed to capture emerging trends and technologies not yet represented in peer-reviewed literature. Articles were selected based on their relevance to one or more of the following themes: (1) AI systems currently implemented or under investigation in oncology and cardiology; (2) Technical, ethical, and clinical challenges associated with AI integration in these fields; (3) Potential opportunities for cross-disciplinary innovation between oncology and cardiology. Non-English articles, conference abstracts, and gray literature were excluded. Because this was a narrative review, no formal quality assessment or meta-analysis was performed. Instead, evidence and perspectives were synthesized qualitatively to highlight conceptual advancements, practical applications, and gaps warranting further investigation.

## 3. AI in Clinical Diagnostics

Deep learning models now detect early signs of cancer and cardiovascular anomalies, two of the leading causes of death globally [3,4], with performance comparable to experienced clinicians [3,17]. The extent, complexity, diversity and size of today’s clinical datasets are all rising [20]. They include unstructured data like radiology reports, pathology images, and free-text clinical notes, as well as structured sources including laboratory results and vital signs [21,22]. DL-based computer vision methods (for classification, detection, segmentation) interpret complex medical imaging [23,24,25], while ML-driven natural language processing (NLP) extracts insights from unstructured text [1,18].

These datasets frequently contain high-dimensional inputs, such as wearable biosensor signals, proteomics, and genomics, that require sophisticated processing and interpretation tools [2]. AI uniquely positions itself to gather valuable insights from a wide range of sources by identifying subtle patterns invisible to the human eye. For example, AI identifies distinct combinations of imaging characteristics, clinical symptoms, and genetic variants that collectively forecast early-onset disease or treatment response [26,27,28].

### 3.1. AI in Laboratory Medicine

Early detection of cancer and cardiovascular diseases improves clinical outcomes. Biomarkers, ranging from genetic mutations to protein and metabolite levels in serum and plasma, play a central role as measurable biological indicators and are among the most effective instruments for monitoring disease presence, progression, and treatment response [29,30]. In oncology and cardiology, key biomarkers include changes in DNA methylation, genetic mutations, and protein expression. They facilitate early disease detection, inform treatment selection, and allow for longitudinal monitoring of therapeutic efficacy. Given the complexity and volume of data involved, especially in multi-layered omics approaches, traditional analytical tools often reach their limits [31]. Over the last three years [32], AI has revolutionized the process of identifying biomarkers by simplifying the analysis of high-dimensional, multi-layered omics data, encompassing genomics, transcriptomics, proteomics, metabolomics, and epigenomics [33]. While traditional single-omics analysis approaches have shown success, they struggle to capture the full heterogeneity of the disease. Recent research is progressing toward multi-omics integration, which involves the simultaneous analysis of multiple omic layers to achieve more accurate and biologically meaningful insights. AI, particularly deep learning models, is well-suited for such tasks since these algorithms can model non-linear relationships, combine different types of data, and detect subtle biological signals [34,35]. For instance, integrative AI models have been applied to glioblastoma subtyping, combining gene expression, DNA methylation, and miRNA data to identify cell clusters with distinct survival patterns and molecular pathways [36,37,38]. Similarly, in breast cancer [39], multi-omics fusion models have demonstrated improved prognostic accuracy compared to models that rely on single data types. These advances demonstrate the life-changing potential of AI-driven multi-omics approaches in precision medicine.

Recent advances in ML have revolutionized cancer prognosis by enabling the integration of diverse biological and clinical data. ML algorithms such as random forests and DL architectures such as autoencoders and multimodal neural networks are increasingly applied to predict long-term survival outcomes with high accuracy [40,41,42,43]. By combining RNA-seq, DNA methylation and clinical features, these models capture complex, non-linear relationships across molecular layers, offering a more holistic view of tumor biology. In colorectal and lung cancer, such integrative approaches have demonstrated significant improvements in predicting five-year survival rates, paving the way for more personalized and data-driven treatment strategies [44]. These examples suggest that these models can serve as general-purpose engines for linking biological signals to clinical outcomes [43].

Modern omics technologies generate vast high-dimensional datasets across diverse platforms, each with its own preprocessing pipeline. This variability introduces batch effects and inconsistencies that obscure biological signals and hinder downstream analysis [45]. To overcome these challenges, AI-driven approaches such as transfer learning, domain adaptation, and data harmonization are increasingly being deployed to align and refine omics data for clinical use [46,47]. Similarly, in cardiology and oncology, multimodal FMs that unite electrophysiology [15], spatial transcriptomics, histopathology, and genomic data may help reveal risk factors tumor heterogeneity and treatment vulnerabilities. By combining information from various modalities, Foundation Models gain a comprehensive understanding that yields better results than using any single modality alone [48]. Digital pathology employs machine learning models to analyze histological and cytological slides, demonstrating a promising application of AI [49,50]. These systems support tumor detection, tissue classification, and prognostic modeling, thereby offering improved efficiency and reproducibility in diagnostic workflow. By leveraging standardized imaging protocols and curated datasets, digital pathology demonstrates how AI can enhance clinical decision-making beyond omics-based data [51,52].

Another intriguing and promising application is the growing use of digital tools in diagnostic laboratories, particularly for cytological screening such as PAP smears. Diagnostic laboratories employ AI-based systems to automatically screen slides, releasing clearly unremarkable findings without manual review. This approach significantly reduces turnaround time, saves personnel resources, and lowers diagnostic cost while maintaining high diagnostic reliability. It exemplifies how AI can streamline routine workflows and support scalable, quality-assured diagnostics in real-world clinical settings.

Despite these advances, reliable recognition of meaningful patterns remains a hurdle. Interpretability is another critical bottleneck. Many deep learning models operate as “black boxes”, complicating clinical validation and slowing regulatory approval [53]. On one hand, the issue has led to increased interest in explainable AI (XAI) approaches, including attention-based models and explainable boosting machines, which provide transparency by highlighting the most influential features across omic layers. On the other hand, there is an urgent need for standardized clinical omics workflows. These must include automated, reproducible pipelines supported by robust and reliable quality management systems to ensure consistency and trustworthiness of clinical data. The successful integration of multi-omics and AI into clinical practice will hinge on several key developments: (1) the creation of interoperable omics databases, (2) standardization of preprocessing protocols, (3) rigorous model validation across multi-center, multi-ethnic cohorts, and (4) a strong commitment to fairness, interpretability, and readiness for regulatory approval.

### 3.2. AI and Imaging-Based Applications

AI applications are increasingly applied to imaging modalities such as positron emission tomography (PET), echocardiography, CT angiography, MRI, and optical coherence tomography (OCT), to streamline workflow, minimize interobserver variability, and enhance clinical decision-making [54]. Radiological imaging techniques remain the gold standard in cancer diagnostics, specifically for delineating tumor boundaries and assessing the therapy response [55]. Tumor semantic segmentation methods offer rapid and accurate determination of the diseased tissue by performing a pixel-by-pixel analysis and informs radiotherapy strategies, surgical navigation and tumor recurrence [56]. CNNs have remained the focus of tumor segmentation for a long time due to their effectiveness in detecting tumor boundaries and tissue heterogeneity [57]. This success highly depends on convolutional kernels that can capture local spatial features and architectures such as U-Net and their variations achieve precision in feature extraction [58]. CNN variants also allow 3D analysis and measures the volumetric changes, which are essential to observe tumor growth. Yet, CNNs are limited to grasp long-range dependencies across an image where tumors can present with irregular shapes, or scattered lesions [59]. To address this challenge, researchers have increasingly turned to transformer-based models. Vision transformers and hybrid transformer-CNNs utilize self-attention mechanisms, so that the model can capture global contexts while retaining the local details [10,60]. This is done using transformer blocks that connect the information between distant tumor regions and surrounding healthy tissue, offering better segmentation outcomes. Today, hybrid transformer-CNN approaches rely on CNN layers for low-level feature extraction and transformers for high-level interactions [60]. This approach has recently shown promising results in the brain tumor segmentation challenge where hybrid methods identified multiple tumor subregions including enhancing tumor and tumor core [61]. Recent work by Neha et al., 2025 has provided a comprehensive analytical comparison of the U-Net architecture and its major extensions, including U-Net++, U-Net 3+, and hybrid Transformer–U-Net frameworks [62]. This review systematically examined how architectural refinements such as nested and full-scale skip connections, attention gating, and deep supervision have enhanced segmentation accuracy, generalizability, and computational efficiency across modalities such as MRI, CT, ultrasound, and X-ray. The study also highlighted the clinical significance of these models in improving delineation of tumors, myocardial structures, and vascular regions, emphasizing their pivotal role in oncology and cardiology imaging.

Foundation segmentation models, including the Segment Anything Model (SAM) [63] and medical adaptations such as MedSAM [64] and SAM-Med2D [65], have introduced zero-shot generalization, reducing annotation burden and enabling rapid cross-modality transfer across MRI, CT, and pathology data. To enhance clinical interpretability, explainable AI (XAI) tools like Grad-CAM and attention heatmaps are increasingly integrated, allowing radiologists and pathologists to validate model focus and build trust in automated outputs. These achievements also increase the model generalizability across modalities and patient populations, bringing us one step closer to clinical adoption [10].

Among image-based AI applications, skin cancer serves as an example where AI has demonstrated strong potential for accurate disease detection while also highlighting the risks involved. Researchers demonstrated [66] that by training a CNN, the model matched dermatologist level of accuracy in classifying benign and malign skin cancers but the training for the model was done mainly on images of light skin [66]. Once applied to patients with darker skin tones, these models show a marked drop in diagnostic accuracy, increasing the risk of delayed detection and poorer outcomes. This example highlights how imbalanced training datasets can amplify health disparities if models are deployed without robust external validation and fairness assessment [67].

**Figure 1:**
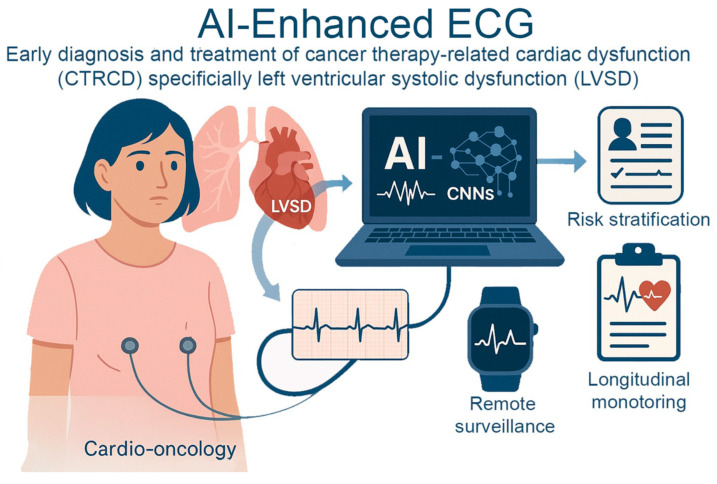
**AI-Enhanced ECG for Early Detection of Cancer Therapy–Related Cardiac Dysfunction.** AI-enhanced electrocardiography in cardio-oncology identifies cancer therapy–related cardiac dysfunction, specifically left ventricular systolic dysfunction. AI models using convolutional neural networks analyze subtle ECG changes to detect early myocardial damage before clinical symptoms arise. This enables proactive risk stratification, remote surveillance through wearable technologies, and longitudinal monitoring.

In cardiology, one review explored how AI-driven echocardiography is speeding up workflows and reducing measurement variability in cardiovascular diagnostics through fully automated segmentation, view classification, and automatic measurements of ventricular/atrial volumes, ejection fraction and strain [68]. Commercial tools are available [69,70,71] and can generate guideline-based diagnostic reports. The American Heart Association supports the implementation of these tools within clinical infrastructure, emphasizing their potential to reduce disparities in cardiovascular care and support outcome-driven risk modeling [20]. Most importantly, studies have proven increased reproducibility across various skill levels of physicians. However, AI’s accuracy still relies on high-quality imaging and proper anatomical identification, indicating some limitations (Figure 1).

Furthermore, AI models, especially deep learning approaches, have achieved high accuracy (AUCs > 0.9) in ECG interpretation and detecting arrhythmias, including atrial fibrillation (AF) [72,73]. In several studies, AI models either matched or surpassed the diagnostic accuracy of cardiologists and other physicians in diagnosing rhythm disorders [74,75,76]. These models are able to extract subtle variations in signals from baseline that exceed the capability of human observers. Additionally, it has been shown that a simple tool such as ECG, integrated with clinical history can improve the prediction accuracy of AI models for clinical outcomes or even mortality risk [26]. While there are available tools integrated to enhance ECG and Holter readings, there seems to be a lack of tools that assess stress tests. A study showed that machine learning might outperform cardiologists in predicting the presence of stress-induced functional coronary artery disease (AUC for ML 0.71 vs. AUC for cardiologist 0.64, *p* = 4.0 × 10^−13^) [77].

**Figure 2:**
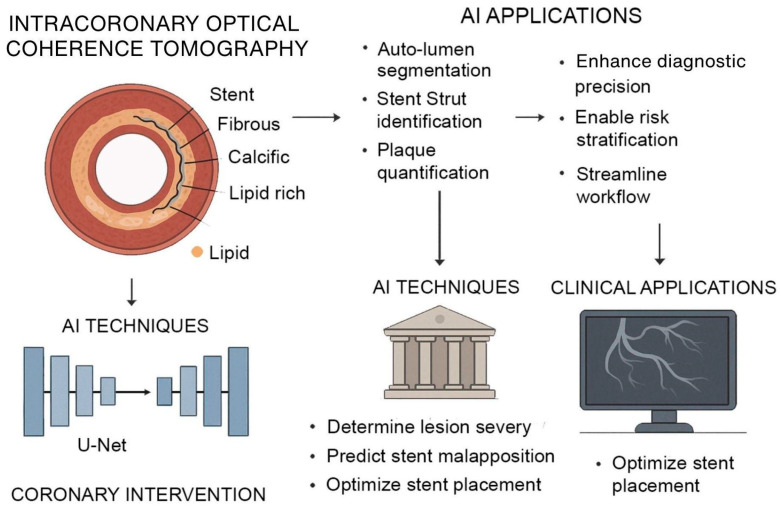
**AI-Augmented Intracoronary OCT for Coronary Intervention.** AI enhances intracoronary OCT for improved coronary assessment and intervention. OCT provides high-resolution cross-sectional imaging of vascular structures, enabling characterization of fibrous, calcific, and lipid-rich plaques. AI models, particularly U-Net architectures, support automated lumen segmentation, stent strut identification, and plaque quantification. These techniques assist in determining lesion severity, predicting stent malapposition, and optimizing stent placement.

As AI methods continue to mature, its integration with advanced imaging platforms is significantly transforming intercoronary OCT, a high-resolution imaging modality critical for assessing atherosclerotic plaque morphology and guiding interventional procedures. Traditional OCT analysis requires expert interpretation of thousands of images, a process that is time-consuming and sensitive to variability. Deep learning–based frameworks now enable complete-vessel segmentation, automated lumen border detection, stent strut identification, and volumetric plaque quantification directly from OCT datasets [78,79,80]. These models, such as U-Net architectures trained on expert-annotated datasets, have demonstrated pixel-level accuracy in classifying fibrous, calcific, and lipid-rich plaque with high reproducibility, enabling real-time decision support during percutaneous coronary interventions. This capability helps interventional cardiologists determine lesion severity, predict stent malapposition risk, and optimize stent placement (Figure 2).

Recent studies have explored the integration of advanced technologies into AF ablation strategies to improve precision. Deisenhofer et al. (2025) [81] demonstrated that real-time tagging with AI and tailored ablation is feasible, operator-independent, safe, and precise. The TAILORED-AF randomized controlled trial showed that combining AI-guided DISPERS mapping with pulmonary vein isolation significantly improved 12-month AF-free survival (88%) compared to pulmonary vein isolation alone (70%), with strong statistical significance (*p* < 0.0001). Compared to the standard approach, this method, being more precise, offers a tailored operator-independent approach to AF ablation [81].

In addition to procedural enhancements, advanced imaging and mapping techniques are improving the ability to identify and treat AF triggers. Deep learning applied to cardiac imaging predicted non-pulmonary vein sources with 82% accuracy, aiding in the detection of atypical foci. Enhanced ECG-based mapping tools have also helped reduce ablation time and increase procedural efficiency. Together, these innovations support a more personalized and effective approach to AF treatment, moving beyond conventional ablation strategies [82].

### 3.3. Excursus-AI and Voice Biomarkers

Recent AI innovations have enabled the extraction of clinically relevant information from non-traditional data sources, including the human voice. Researchers have pioneered work on voice-based biomarkers, where acoustic speech features can reflect underlying physiological changes [83,84]. Studies [85,86,87] have demonstrated that machine learning models can detect early decompensation in heart failure patients based on vocal strain, breathiness, and prosody alterations. Up to 80% in detecting decompensation [87], while the prospective acute heart failure-voice study is currently characterizing daily voice fluctuations in hospitalized patients [88]. Beyond heart rate, AI-based speech systems can distinguish “wet” versus “dry” fluid states in hospitalized patients with ~94% accuracy and have been piloted to predict coronary artery disease using smartphone-recorded speech samples [89]. These tools promise continuous, remote surveillance and early risk stratification, supporting proactive intervention and potentially reducing rehospitalization. But it is still a challenge to replicate these findings and to ensure their generalizability [90].

**Figure 3:**
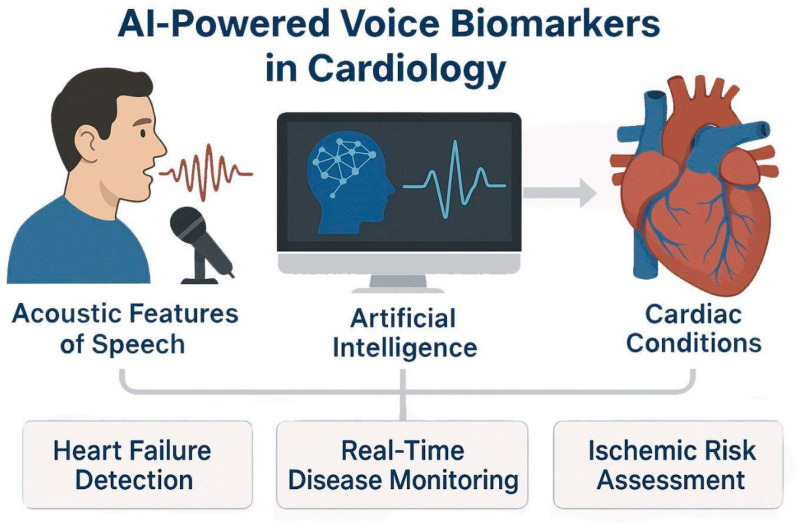
**AI-Powered Voice Biomarkers in Cardiovascular Disease Detection and Monitoring.** AI models analyze acoustic features of speech and extract voice-based biomarkers relevant to cardiology. Using techniques such as speech signal processing and prosodic feature extraction, AI detects physiological changes associated with heart failure, fluid status, and ischemic heart disease.

These systems not only match expert-level performance but are optimized for real-time deployment during catheter-based interventions, enhancing diagnostic precision, streamlining workflows, and supporting interventional decision-making in high-stakes cardiology settings (Figure 3). Despite initial success, the translational maturity of speech biomarkers is hampered by a significant lack of reproducibility. Model performance is highly sensitive to uncontrolled variables, including acoustic recording conditions and linguistic factors, meaning robust clinical utility requires standardized data acquisition protocols that are currently underdeveloped for diverse, uncontrolled patient environments [78,83].

### 3.4. Use Case—Cardio-Oncology with AI-ECG

Cardio-oncology intersects the fields of cardiology and oncology. AI-enhanced electrocardiography (AI-ECG) is one of the most promising applications in this field for the early diagnosis and treatment of cancer therapy-related cardiac dysfunction (CTRCD), specifically left ventricular systolic dysfunction (LVSD). ECG and other traditional surveillance methods frequently fail to identify dysfunction until irreversible myocardial damage has taken place. AI-ECG, on the other hand, offers a non-invasive, scalable alternative that can detect subtle ECG changes indicative of early-stage cardiac damage [91,92].

AI-ECG utilizes CNNs to detect subtle ECG indicators of left ventricular dysfunction (AUC ~ 0.93) [93]. These tools have been applied to patients on cardiotoxic cancer treatments, such as anthracyclines, *HER2*-targeted agents, and immune checkpoint inhibitors [94]. When paired with large language models (LLMs), AI-ECG forms a hybrid pipeline melding signal-based prediction with textual context to support risk stratification, longitudinal monitoring, and early cardio-oncology referrals. Initial data indicate that AI-ECG exhibits high sensitivity and can be conducted in an outpatient environment. This suggests that routine echocardiograms may be less essential and that transfer to cardio-oncology services can be expedited. This integration of multimodal AI, including wearable technology, narrative data, and signal data, represents a significant advancement in precision cardio-oncology [95]. The highly accurate performance of AI-ECG models is often achieved on clean, internal datasets [96]. However, these models exhibit limited generalizability in the real world due to data shifts caused by variances in ECG machine manufacturers and protocols across different hospital systems [97]. This susceptibility calls for extensive and costly large-scale prospective validation before clinical integration. Continuous validation efforts are essential to ensure that these novel concepts are applicable in clinical environments, scalable and evidence-based. As AI-ECG systems advance, they may become crucial tools for predicting and mitigating cardiotoxicity induced by cancer therapies.

## 4. AI in Clinical Trials and Real-World Evidence

Clinical trials, particularly those in oncology and cardiology, are a crucial component of medical progress; however, they face ongoing and increasing challenges that hinder their efficiency, scalability, and overall benefits [98]. These trials are often too expensive and it can take more than ten years for the drugs to get regulatory approval. The slow progress is exacerbated by numerous operational issues, including slow patient recruitment, high dropout rates, and data being dispersed across disparate electronic systems that do not integrate effectively. EHRs, imaging archives, and wearable devices often house trial-related data in separate systems. Such separation makes it difficult to combine the data and analyze it in real time. Adding RWE is beneficial, but it introduces more complexity because it is not structured and does not have standard formats. The choice of location is a critical issue, and decisions are often based on past relationships or static feasibility studies instead of real-time data on capacity, infrastructure, or patient demographics. These inefficiencies limit therapy outcomes and contribute to the high failure rates and cost overruns that are common in drug development today [99].

On the largest primary clinical trial registry worldwide, there are currently more than 3000 [100] registered clinical trials that include the mesh terms ‘artificial intelligence’ or ‘machine learning.’ AI can enhance nearly every aspect of the trial lifecycle by leveraging machine learning models, natural language processing, and multimodal data integration. AI systems allow for a more responsive and patient-centered approach, from smart protocol design and eligibility refinement to adaptive dosing schedules and real-time safety monitoring.

Furthermore, moving beyond trial optimization, AI demonstrates clinical utility in large-scale screening. For instance, AI-enabled ECG screening for low ejection fraction exemplifies robust methodological rigor, as it evaluates screening utility in routine practice, shifting the focus from simple diagnostic accuracy to public health impact. In this use case, AI-enabled ECGs increased the diagnosis rate of low ejection fraction in routine practice (e.g., from 1.6% to 2.1% in the intervention arm [101,102].

Similarly, in oncology, large-scale prospective breast cancer screening trials are implementing AI tools directly into the live screening process and comparing them against the standard of care. These studies, involving hundreds of thousands of women, are designed to assess whether AI can help radiologists to detect breast cancer cases earlier while reducing the reading burden by speeding up the detection process and enhancing the patient-level benefit by earlier diagnosis. Data quality remains a central limitation in AI-driven trials. LLMs and emerging tabular foundation models, such as TabPFN [103], offer new avenues for automating data cleaning, harmonizing multimodal inputs, and augmenting prediction performance in small or fragmented datasets, thereby improving both model robustness and translational potential.

For example, platforms like Tempus are already being used in cancer trials to combine genetic information, medical records, and real-life data to identify specific groups of patients who are most likely to benefit from immunotherapies, such as PD-1/PD-L1 checkpoint inhibitors in non-small cell lung cancer. These tools also improve treatment planning for early-stage breast cancer by improving models that predict the risk of recurrence based on traditional tests like Oncotype DX. The EVIDENCE-HF trial shows how AI-powered remote monitoring, along with wearable sensors and EHR data, can predict decompensation in heart failure patients. This lets physicians intervene early and reduces data loss due to dropouts. These insights can also aid in adaptive trial designs, which adjust the dosing schedule or visit intervals based on changes in a patient’s risk profile over time [104]. Despite these promises, a clinical trial tool that aimed to enroll patients based on sequencing data utilizing automated clinical trial notifications showed limited impact and adoption. Half of the oncologists receiving the notifications did not engage with them and incorporate them in their clinical workflow. Moreover, patients chose not to enroll with specific trials recommended by the AI and preferred other options, highlighting the importance of clinical acceptance for AI tools by both the clinicians and patients [105].

### 4.1. AI-Driven Innovations

**Figure 4:**
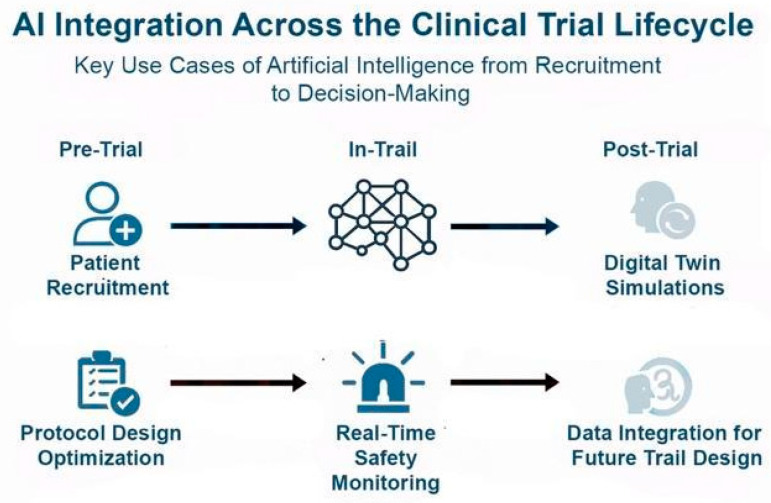
**AI Integration across the clinical trial lifecycle:** In the pre-trial phase, AI optimizes patient recruitment and protocol design. During trials, AI enables real-time safety monitoring, improving responsiveness to adverse events. Post-trial, AI contributes to digital twin simulations and integrates clinical and real-world data to inform future trial design. AI leverages tools like NLP to extract insights from unstructured data (e.g., physician notes, patient comments), enhancing Phase IV studies, pharmacovigilance, and long-term efficacy assessment in real-world settings.

Beyond their role during clinical trials, AI tools can help understand the complexities of real-world data obtained after approval and aid in shaping the clinical landscape. These aids can include filling the electronic health records, a task that is highly repetitive and challenging, which often leads to missing data that are crucial for patient management. It is also of the challenges for creating reliable datasets which are essential for the next generation of clinical AI models. AI can also utilize unstructured texts such as doctors’ notes and other materials and incorporate them in the real-world data using NLP. Moreover, AI models can identify adverse events, patients’ comments, and other clinical findings post-drug approval and utilize them for their Phase IV studies or to assess the clinical efficacy of their drug [106]. Below, we showcased two examples of how a combination of AI with diagnostics and real-world data can lead to significant advances in clinical trials (Figure 4).

### 4.2. Use Case: Digital Twin

A digital twin is a virtual representation of a patient created using real-world medical data, including electronic health records, laboratory results, genetic information, and data from wearable devices. In contrast to conventional simulations, which are static snapshots that depict prescribed scenarios under specific conditions, a digital twin is a living representation of a specific physical asset. It is constantly updated with real-time data, enabling an ongoing comprehension of a patient’s health. These models enable researchers and clinicians to simulate the potential responses of individuals to various treatments, medications, or health conditions without compromising the safety of actual patients [29]. This innovation brings clinical trials closer to the promise of precision medicine, in which interventions are customized to the unique biology and clinical history of each individual [107,108].

**Figure 5:**
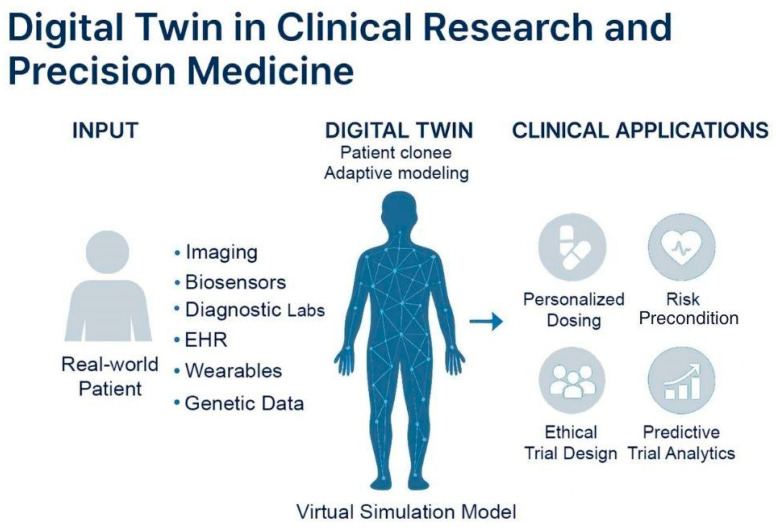
**Concept and clinical applications of digital twin models.** Virtual representations of real-world patients continuously updated with multimodal health data, including imaging, biosensors, diagnostics, EHRs, wearables, and genetic information. These models simulate patient-specific physiology using adaptive algorithms to guide personalized care. Digital twins support precision medicine by personalized dosing, risk prediction, predictive trial analytics, and ethical trial design.

In real-world settings, digital twins are already being piloted in specialized areas to simulate disease progression, optimize treatment planning, and reduce the burden on traditional trial infrastructure. For example, at the Gustave Roussy Cancer Campus in France, researchers have deployed digital twin models for patients with metastatic breast cancer to simulate tumor progression and evaluate various chemotherapy regimens. These simulations enabled clinicians to forecast treatment response over 6–12 months, optimizing both dose and timing in a patient-specific manner without real-world exposure to toxicity. Similarly, the “SimCardioTest” project, funded by the European Commission, uses digital twins to simulate cardiovascular responses to antiarrhythmic drugs across diverse virtual populations. By incorporating electrophysiological models and patient-derived ECG data, these twins help predict QT prolongation and arrhythmia risk prior to human trials, reducing adverse event rates. Digital twins also support broader participation in clinical research by adjusting treatment protocols for higher-risk individuals who might otherwise be excluded under rigid eligibility criteria. In cases of participant withdrawal, digital twins can continue simulations using previously collected data, preserving statistical power and reducing attrition bias. This enhances the robustness of trial outcomes and minimizes data loss [29]. Beyond the scope of clinical trials, digital twins continue to offer utility in real-world clinical practice. They can help clinicians simulate patient-specific responses to a range of treatment options, including variations in dosage, timing, and sequencing, without requiring additional invasive procedures (Figure 5). This is particularly valuable in complex therapeutic areas such as oncology, where treatment decisions often involve a balance between efficacy and toxicity. While conceptually powerful, current evidence is rooted in single-center pilot simulations [109]. Digital twins face severe scalability challenges due requiring massive, high-dimensional, and continuously updated datasets, which fundamentally limits reproducibility and compromises external validity outside of heavily resourced academic medical centers [110]. As post-approval real-world data accumulates, digital twins will improve in accuracy, providing clinicians with continuously updated guidance that reflects the latest evidence and population-level insights.

### 4.3. Use Case: Data Extraction from Unstructured EHRs

EHRs comprise significant portions of healthcare data, but this vast amount of knowledge was locked within the unstructured text [106]. Handwritten notes, physician dictations, and diagnostic reports are all critical details about a patient’s journey, spanning from symptom onset to treatment journey. Given the labor-intensive and error-prone processes required, manual extraction of these data remained challenging [111]. Clinical AI, particularly through NLP applications, hold the potential to systematically extract these data with high accuracy and transform the clinical practice into data-driven medicine.

AI can bridge the gap between humans and structured, quantifiable data while NLP algorithms parse unstructured free-text clinical notes to obtain key information [106]. As the AI algorithms communicate, they can identify and prioritize relevant information and bring it to the attention of the healthcare personnel. For example, clinical AI models can extract patients’ comorbidities and their current medications as well as their medical history. This approach allows clinicians to access not only demographic data but also the patients’ health journey. As unstructured texts become structured databases, large-scale analysis of patient populations and clinical trends becomes more apparent, offering a foresight for clinical trends [112].

AI-powered data extraction from EHRs offers to transform our current medical practices in the near future [111]. Using EHR with powerful algorithms, we can query the hospital records in a few minutes to match complex inclusion criteria with patients and create specific cohorts for clinical trials. These data also offer novel approaches to personalized medicine, as AI can identify risk factors and offer treatment approaches unique for each individual. In the end, AI will serve as a co-pilot of the physician as it performs administrative and repetitive tasks while allowing the doctors to focus on patient care.

## 5. Challenges and Barriers to Clinical AI Integration

Despite the increasing innovation in AI applications in oncology and cardiology, real-world clinical integration remains limited due to several challenges. A central issue is the occurrence of hallucinations and overall accuracy of AI tools that suffer from false positives and overfitting. Optimizing AI systems on narrow datasets often leads to results that are not reproducible across clinical sites or broader patient populations [113]. In high-stakes settings, such as early cancer detection or acute cardiovascular interventions, even a small margin of error can have significant clinical and psychological consequences. For instance, a false-positive cancer diagnosis can lead to unnecessary interventions and could result in patient anxiety and even invasive procedures. AI-assisted diagnostics can improve long-term survival by enabling earlier, more accurate detection of diseases like cancer or heart failure [114,115,116]. However, its effect on patient experience remains mixed. Studies show that patient trust often declines when they are explicitly aware that a physician is using AI, driven by concerns over data privacy, a perceived reduction in human empathy, and the potential for a less personal relationship [117,118,119]. The overall impact hinges on transparent communication and the successful integration of AI as a supportive tool that augments, rather than replaces, the essential human element of healthcare [120]. In addition, the impact of implementing AI in hard outcomes such as mortality remains to be elucidated as large, long-term trials are missing.

Beyond accuracy and reproducibility, practical integration into existing clinical workflows is another barrier. For AI systems to be useful, they must fit seamlessly into EHRs and clinical decision-support systems that require significant investments in infrastructure and training. While regulation of medical devices and pharmaceuticals are well established, harmonized regulatory frameworks are still being determined for AI-based tools. The absence of globally accepted standards for model validation, continuous monitoring, and post-deployment surveillance makes it difficult for clinicians and health systems to trust AI applications at scale [121].

This lack of harmonized regulatory frameworks and validation standards creates a critical synthesis point: the “Black Box” dilemma. High-performance deep learning models often lack the necessary Explainable AI to justify their output, which directly undermines clinical trust and impedes regulatory approval as a software as a medical device. Without transparent mechanisms showing how a model arrived at an oncological or cardiovascular decision, neither the clinician nor the regulator can ethically accept accountability. Technical limitations such as poor data quality interact with ethical concerns like bias propagation, all of which are ultimately compounded by operational barriers related to clinical workflow and economic feasibility. A true synthesis requires understanding this interplay, as performance on a clean, single-center dataset, for instance, provides little insight into a model’s operational viability or regulatory readiness.

The pathway to integration must therefore be comprehensive and end-to-end, beginning with the identification of clinically relevant problems, followed by dataset selection, model development, and rigorous validation, as well as ethical and legal evaluations. In the sections below, we offer several approaches to these problems that would aid the adoption of AI in clinical settings.

### 5.1. Validation, Regulation, Explainability

AI models can reveal new insights from large datasets, but poor data quality or biased training can lead to errors. Validation against established clinical evidence remains essential to ensure reliability. Frameworks like the 24-step guide proposed in [122] outline best practices for protocol registration, bias assessment, and transparent reporting. These are highly applicable to validating AI systems with real-world clinical data. When an AI model offers insight, it is essential to confirm findings through case studies as well as retrospective or prospective clinical evaluations. Training data must also be representative, diverse, and high-quality, with attention to population demographics and longitudinal consistency [123].

Model validation should encompass multiple dimensions: predictive accuracy, calibration, robustness to missing or noisy data, and clinical interpretability. Benchmarking against traditional statistical methods and evaluating performance in live or retrospective trial settings are critical. Validation practices should also include ethical reviews, regulatory alignment, and mechanisms for continuous improvement through real-world feedback. Beyond validation, interpretability is equally critical. As AI becomes more deeply integrated into clinical trials and decision-support systems, XAI provides transparent, interpretable, and clinically meaningful outputs [124].

XAI methods offer both global and individual-level explanations. For example, a model predicting survival after surgery may highlight age and comorbidities as global predictors, while identifying personalized risk factors for an individual patient. Some models are inherently interpretable by design, offering a direct window into decision-making. Examples of such “glass-box” approaches include generalized additive models [106,125] and explainable boosting machines [126,127]. These models show the contribution of each feature to a prediction, often through intuitive shape functions clinicians can easily visualize. For instance, predicting disease risk might show how increasing patient age or a specific lab value monotonically increases risk, while another feature may have a more complex, non-linear relationship. This transparency lets clinicians inspect how patient characteristics contribute to outcomes, fostering trust without complex post-processing. In image-based AI (e.g., histopathology), integrated gradient techniques can show which regions of a scan influenced the diagnosis. In dynamic clinical contexts, time-series XAI methods help practitioners understand how trends in vital signs or lab values affect outcome predictions, especially in perioperative or intensive cure unit settings. Similarity classification techniques further enhance trust by comparing current patients to past cases and illustrating the accuracy of prior predictions.

These tools empower clinicians, support patient communication, and aid regulatory review. Recent advances, such as the integration of LLMs [106] and visual language models, extend these principles into multimodal and conversational interfaces, making it easier for clinicians to interact with AI systems via natural language or multimodal inputs. Embedding explainability into AI tools ensures clinicians remain in control, fosters transparency, and enhances collaboration between clinical and technical stakeholders, an essential step toward widespread and responsible AI adoption in healthcare [128].

### 5.2. Data Harmonization

Real-world data consists of many stakeholders, which can include wearable device companies with health monitoring data or hospitals with electronic health records. This type of standardized quality monitoring ensures that models trained on multicenter datasets are interoperable and generalizable [129]. Furthermore, data harmonization is inextricably linked to the ethical hurdle of algorithmic bias. Datasets that lack representativeness (e.g., limited to a single ethnicity or socioeconomic group) will inevitably lead to models that propagate and amplify existing health disparities when deployed in diverse populations. Therefore, the technical synthesis of large, diverse, and interoperable datasets is a prerequisite for achieving ethical and equitable AI translation.

It is also challenging to bring together various parties and promote data sharing, as each party might want to retain control or extract value from the data it holds. Here, the stakeholders can adopt a collaborative approach and form consortia with a commitment to share data with one another. Health data integration at such large scales can result in life-changing insights and interventions for the patients. When datasets are combined, which include patients’ electronic health records and wearable device data, patient-identifying information must be removed before training the model. Handling such sensitive information requires attention, secure databases, and reliable personnel. This demands data custodians, such as hospitals and research centers, that can gather all this information while ensuring patient privacy.

## 6. Enabling Responsible AI

The successful application of AI in clinical trials requires both data harmonization and interdisciplinary collaboration along with trustworthiness. Real-world data from hospitals, wearables, and pharmaceutical research must be made interoperable through standardized formats, ontologies, and metadata schemas. Secure data-sharing frameworks and collaborative governance structures are essential for preserving patient privacy while enabling innovation. Trustworthiness includes strong predictive performance, calibrated uncertainty quantification, robustness to distribution shift, and out-of-distribution detection, all of which safeguard clinical reliability [130,131]. Training programs should incorporate both technical and clinical curricula, promoting collaborative environments that develop AI applications with clinical relevance, statistical rigor, and regulatory compliance [1]. Together, these frameworks ensure that AI augments current practices in a manner that is trustworthy, equitable, and sustainable across the healthcare ecosystem.

## 7. Pitfalls and Limitations of Artificial Intelligence

Despite these promises of AI in advancing cardiovascular and cancer care, its responsible and effective implementation necessitates overcoming critical issues. A key challenge is to ensure the data quality and representativeness. Given that the model’s performance is determined by both the quality and quantity of the training data, only datasets that include diverse clinical diseases and ethnicities can offer AI tools that can capture the complexity of the clinical space [132,133,134]. For example, recent AI-based imaging tools developed on single-center datasets have shown reduced diagnostic accuracy when externally validated, revealing how limited data diversity can undermine real-world performance [135]. This challenge is especially relevant for LLMs, clinical agents, and multimodal foundation models, which necessitate extensive, high-quality datasets to prevent the propagation of bias or the generation of false correlations. Hallucinations, fabricated but plausible-seeming outputs, are a well-documented risk for LLMs, especially in safety-critical tasks such as clinical summarization or decision support. Such datasets should include patient characteristics such as age, sex, socioeconomic background and comorbid conditions to improve model generalization, accuracy, and reliability across the population, which can otherwise lead to biased outcomes and reduce clinical trust. At the same time, as AI technologies gain popularity, they also raise significant issues such as data ownership, privacy and data security [130,131,136]. Compliance with the data protection regulations in each country requires local deployment of AI models that need to be embedded within the IT infrastructures of healthcare providers. Given their previous experience with sensitive data, such institutions, rather than external cloud-based companies would better ensure the protection of patient populations. The compliance would also allow the institutions to extract meaningful clinical insights from unstructured data such as clinical notes without compromising privacy.

Beyond data limitations in high-income countries, a more profound gap exists globally [137,138]. While algorithmic bias is frequently discussed in terms of underrepresented racial and ethnic groups in Western contexts, a critical disparity arises because datasets are overwhelmingly derived from high-income countries. This leads to a severe underrepresentation of populations from low- and middle-income countries. This disparity ensures that models are trained on disease profiles, comorbidities, and imaging standards that may be irrelevant to the clinical reality of low- and middle-income countries [137]. Consequently, AI solutions designed for high-income countries may amplify global health inequity by failing to offer accurate, accessible, and affordable diagnostic tools where they are needed most. This lack of diverse geographic data directly correlates with systemic disparities in access to trustworthy, validated AI technology [138]. Yet another limitation of AI tools is their lack of robust validation frameworks. Several prototypes have emerged from academic literature, but few have gone through rigorous and multi-phase clinical validation necessary for regulatory approval and widespread adoption. Moreover, even FDA-cleared AI algorithms often lack long-term post-market validation, and their generalizability across institutions remains limited [139]. Factors that prevent clinical trust include lack of clear criteria for performance benchmarking, clinical efficacy, post-market surveillance strategies, and safety which should be undertaken by regulatory bodies and scientific societies similar to regulations for drug approval and medical devices [140,141,142].

Economic concerns regarding AI tools play a significant role in the implementation of AI. While AI is often referred to as a cost-saving solution in clinics, leading to faster diagnoses, its implementation would at first require significant expenditures and capital investments. Each adoption requires its integration into existing hospital systems, deploying infrastructure such as high-performance local servers, and training the healthcare personnel, and ensuring data privacy along with regulatory compliance [143,144,145].

Several AI tools also require specialized equipment, such as high-resolution scanners, to ensure data quality and also demand recurring software licensing fees [144]. Nevertheless, once integrated, AI offers several long-term economic advantages, such as reducing diagnostic errors, streamlining patient care, and leading to improved performance of clinical staff. Published cost-effectiveness analyses of AI systems in cardiology or oncology are still scarce, and real-world savings have yet to be consistently demonstrated [146]. AI’s role in early disease detection or treatment strategies can lead to timely treatments with improved efficacy, decreasing the clinical burden and reducing the cost of chronic disease management [143,144,147]. As the clinical efficacy of AI is being evaluated, its economic advantages will ultimately determine its adoption; otherwise, it may face resistance from insurance companies and other stakeholders.

Future validation frameworks must evolve beyond conventional performance metrics such as accuracy and AUC toward measures that reflect clinical reliability, uncertainty, and factual integrity. We propose establishing standardized validation metrics across oncology and cardiology that assess both predictive calibration, uncertainty estimation and semantic correctness. For predictive and risk-stratification models, Expected Calibration Error (ECE) [148] should be reported alongside complementary measures such as the Brier score, negative log-likelihood (NLL), and predictive entropy [149], all of which quantify the alignment between predicted probabilities and observed outcomes while capturing uncertainty in probabilistic outputs. Uncertainty estimates expressed through confidence intervals, Monte Carlo dropout variance [150], or ensemble-based predictive distribution [151,152] are crucial for identifying when models “know what they do not know”, thereby supporting clinician oversight. For imaging-based and generative AI systems, domain-specific factuality metrics are equally important. The RadGraph-F1 metric [153] evaluates whether generated radiology or pathology reports accurately capture entities and relationships such as tumor characteristics or cardiac valve abnormalities, while FactScore [154] measures factual correctness and logical consistency in large language model (LLM) outputs. Together, these complementary metrics, covering calibration, uncertainty, and factuality, enable a modality-agnostic validation framework that can unify the assessment of predictive, imaging, and generative AI systems across oncology and cardiology. Such an approach shifts validation from isolated technical performance toward clinically meaningful, interpretable, and trustworthy evaluation standard [20]. Looking ahead, foundation models such as GPT-4o, Med-PaLM 2, and DeepSeek-Med [155,156] may unify these metrics by enabling reasoning across imaging, text, and omics data; however, challenges of interpretability, data provenance, and regulatory validation must be addressed before such systems can be safely deployed in clinical practice.

In conclusion, AI’s impact on healthcare requires beyond technical competency but also equity, privacy, validation, and economic sustainability. Simultaneous development of several AI tools necessitates a reliance on companies’ solutions for their adoption and implementation.

## 8. Conclusions and Future Directions

This review extends beyond single-domain surveys by offering a cross-disciplinary synthesis of AI applications in oncology and cardiology, demonstrating that both fields tackle architecturally similar problems—such as identifying local pathology within large datasets and developing prognostic models for risk stratification. By merging these domains, we benchmark the maturity of emerging technologies and reveal shared barriers, including data harmonization, regulatory challenges, and validation gaps, while highlighting opportunities for mutual learning to enhance clinical translation.

While previous systematics reviews [157] have often focused on single disease domains or specific modalities, this narrative review fills a critical gap by providing a timely, comparative synthesis of AI applications across oncology and cardiology, two fields with high unmet clinical needs and rapidly evolving data ecosystems. We highlight the role of AI in accelerating emerging technologies while critically examining challenges related to evidence quality, validation, equity, and regulation, with a particular focus on translational barriers such as data interoperability, regulatory approval, and clinical workflow integration. By emphasizing both opportunities and pitfalls, this review offers clinicians, data scientists, and policymakers a roadmap for responsible and scalable AI integration in these high-impact specialties.

AI tools aim to enter clinical space and transform medicine, but their adoption in a highly regulated industry remains a challenge. Next-generation clinical AI will likely be powered by multimodal foundation models integrating genomics, imaging, EHR, and wearable data, offering a unified backbone for diagnostics, prognostics, and clinical trial design. A clinical AI model cannot be meaningfully developed in large data centers using only fast algorithms without access to high-quality, diverse, and clinically relevant data [132,136,147]. To achieve this, datasets from stakeholders such as pharmaceutical and insurance companies, hospitals, patient advocacy groups, and government agencies need to come together.

The development and successful integration of clinical AI systems also requires coordinated collaboration between expert groups. Data scientists often face challenges in interpreting complex healthcare data due to pre-analytical variability, while diagnostic and clinical professionals may lack familiarity with AI capabilities and limitations. This gap demonstrates the importance of cross-disciplinary training programs where diagnostic experts, clinical staff, data scientists, engineers, and epidemiologists can communicate effectively, interpret each other’s findings, and refine output [157,158,159]. Interdisciplinary education and team-based problem solving are therefore foundational to building clinical AI tools that are technically sound, trustworthy, and usable in practice.

As the field moves forward, limiting the hallucinations while increasing the transparency and interpretability of clinical AI tools would lead to increased clinical trust and adoption [160]. As regulatory frameworks adapt to rapidly evolving AI systems, clinicians can consult new standards for the continuous validation, monitoring, and reapproval of models that learn over time. Simultaneously, we must interoperate real-world data from hospitals, wearables, registries, and pharmaceutical research using standardized formats, ontologies, and metadata schemas [144]. Secure data-sharing frameworks and collaborative governance structures are essential for preserving patient privacy while enabling innovation [136,144].

This review aimed to provide an integrative overview of AI applications across oncology and cardiology; however, we acknowledge several methodological limitations. Despite the literature search being conducted through PubMed, our approach was not systematic, as our goal was to illustrate AI’s potential through selected case applications in clinical practice. Therefore, our review may not include all existing evidence, and some significant or recent studies may have been missed, both in terms of potential and methodological considerations. Regardless, the examples discussed in this work reflect key trends and translational challenges that define the current state of clinical AI research.

Finally, we posit that profit-driven perspectives are at risk of undermining public trust in healthcare. As regulatory agencies decide on AI’s role in clinical research, forward-thinking entrepreneurs can set an example by prioritizing transparency, accountability, and reliability [92]. In this era of rapidly improving AI technologies, trust and collaboration remain the most powerful tools. Therefore, the crucial responsibility to ethically apply such technologies for the collective good rests firmly with skilled data custodians.

## Data Availability

No new data were created or analyzed in this study. Data sharing is not applicable to this article.

## Glossary

Glossary of Key Terms
- Artificial Intelligence: A broad field of computer science that focuses on creating systems capable of performing tasks that typically require human intelligence, such as reasoning, problem-solving, decision-making, and language understanding.
- Multi-Omics: Integration of multiple layers of biological data (genomics, proteomics, transcriptomics, epigenomics) for more comprehensive disease understanding.
- Digital Twin: A virtual model of a patient used to simulate treatment outcomes and support decision-making in clinical trials and personalized care.
- AI-ECG: AI-enhanced electrocardiogram interpretation to detect subtle changes predictive of cardiac conditions, including therapy-induced dysfunction.
- Oncotype DX: A genomic test used in breast cancer to predict the likelihood of recurrence and the potential benefit of chemotherapy.
- Natural language processing: Is a branch of artificial intelligence that focuses on enabling computers to understand, interpret, generate and interact using human language. It combines linguistics, computer science, and machine learning to process written or spoken language in a way that is meaningful to both machines and people. NLP is used in a wide range of applications, including speech recognition, language translation, sentiment analysis, chatbots, and text summarization. By analyzing the structure and meaning of language, NLP allows machines to respond intelligently to user input and extract valuable insights from large volumes of text data.
- Machine Learning: A branch of artificial intelligence that enables computers to learn from data and improve their performance without being explicitly programmed. Instead of relying on fixed rules, machine learning systems use algorithms to detect patterns, make predictions, and support decision-making.
- Deep Learning: A specialized subset of machine learning that uses artificial neural networks with multiple layers to model complex patterns in large datasets, often applied in areas such as image recognition, natural language processing, and speech analysis.
- Explainable AI (XAI) refers to a set of methods and techniques that make the decisions and inner workings of artificial intelligence systems understandable to humans. Unlike traditional “black box” models, which can produce accurate results without revealing how they reached those conclusions, XAI aims to provide transparency by explaining why a model made a specific prediction or decision.
- Electronic Health Records (EHRs) are digital systems used by healthcare experts to collect, store, and manage patients’ medical information over time. They include structured data such as diagnoses, medications, lab results, vital signs, and clinical workflows. Unlike patient registries, which are designed for research or public health purposes, EHRs are primarily transactional and visit-centered. However, EHRs can support registries by supplying valuable data. Their integration with registries faces challenges such as data quality, interoperability, and privacy.
- A Large Language Model (LLM) is a type of artificial intelligence designed to understand, generate, and process human language. It is trained on big amounts of text data and uses advanced mathematical structures called neural networks to learn patterns in language, such as grammar, context, and meaning. LLMs can perform a wide range of language-related tasks, including answering questions, summarizing texts, translating languages, writing content, and even generating code. Their ability to produce human-like responses makes them valuable tools in fields like education, research and healthcare. However, because LLMs learn from existing data, they can also reflect biases or inaccuracies found in their training material, which is why responsible use and oversight are important.
- Visual Language Models are a type of AI that can understand and create information using both images and videos as well as text. These models learn from big datasets that include images and their descriptions or captions. This lets them understand visual content and respond in human language. Visual language models can do things like describe pictures, answer questions about visual scenes, make captions, or even make new pictures based on text prompts. They are used a lot in medical imaging, education, robotics, and tools for making things easier to use. They help connect visual perception and language understanding.
- Convolutional Neural Network: A type of deep learning model especially effective for processing grid-like data, such as images. CNNs use convolutional layers to automatically detect patterns like edges, textures, and shapes by scanning small regions of the input. This allows the network to learn spatial hierarchies, where early layers detect simple features and deeper layers capture complex structures. CNNs are widely used in tasks such as image classification, object detection, and facial recognition.
- Artificial Neural Network A computing model inspired by the structure and function of the human brain, made up of layers of connected nodes called neurons. Each neuron receives input, processes it (typically using a weighted sum and an activation function), and passes the result to the next layer. ANNs can learn patterns from data and are used in many tasks like image recognition, language processing, and prediction. They are the foundation of many deep learning models, including CNNs and RNNs.
- Generalized Additive Model: A type of statistical model that extends generalized linear models by allowing non-linear relationships between the predictors and the outcome. GAMs use smooth functions (like splines) to model each predictor’s effect individually, then add them together to predict the response. This makes GAMs useful for uncovering complex patterns in data while remaining interpretable. They are often used in fields like epidemiology, finance, and environmental science.

## Abbreviations

AI	Artificial Intelligence
AF	Atrial Fibrillation
AUC	Area Under the Curve
AI-ECG	AI-enhanced Electrocardiogram
CNN	Convolutional Neural Network
CT	Computed Tomography
CTRCD	Cancer Therapy-Related Cardiac Dysfunction
DL	Deep Learning
ECG	Electrocardiograms
EF	Ejection Fraction
EHR	Electronic Health Record
FDA	Food and Drug Administration
*HER2*	Human Epidermal Growth Factor Receptor 2 Negative
HF	Heart Failure
LLM	Large Language Model
LVSD	Left Ventricular Systolic Dysfunction
miRNA	microRNA
ML	Machine Learning
MRI	Magnetic Resonance Imaging
NLP	Natural Language Processing
OCT	Optical Coherence Tomography
Oncotype DX	21-gene expression test for breast cancer recurrence risk
PD-L1	Programmed Death-Ligand 1
PET	Positron Emission Tomography
RNA-Seq	RNA Sequencing
RWE	Real-World Evidence
Tempus	AI-driven precision medicine platform in oncology
XAI	Explainable Artificial Intelligence

## References

[1] Fountzilas E., Pearce T., Baysal M.A., Chakraborty A., Tsimberidou A. (2025). Convergence of evolving artificial intelligence and machine learning techniques in precision oncology. npj Digit. Med..

[2] der Schaar M., Peck R., McKinney E., Weatherall J., Bailey S., Rochon J., Anagnostopoulos C., Marquet P., Wood A., Best N. (2025). Revolutionizing Clinical Trials: A Manifesto for AI-Driven Transformation. arXiv.

[3] Cardiovascular Diseases. https://www.who.int/health-topics/cardiovascular-diseases.

[4] Cancer. https://www.who.int/news-room/fact-sheets/detail/cancer.

[5] Lim Y., Choi S., Oh H.J., Kim C., Song S., Kim S., Song H., Park S., Kim J., Kim J.W. (2023). Artificial intelligence-powered spatial analysis of tumor-infiltrating lymphocytes for prediction of prognosis in resected colon cancer. npj Precis. Oncol..

[6] Wang S., Yang D.M., Rong R., Zhan X., Fujimoto J., Liu H., Minna J., Wistuba I.I., Xie Y., Xiao G. (2019). Artificial Intelligence in Lung Cancer Pathology Image Analysis. Cancers.

[7] Millward J., He Z., Nibali A., Mouradov D., Mielke L., Tran K., Chou A., Hawkins N., Ward R., Gill A. (2025). Automated deep learning-based assessment of tumour-infiltrating lymphocyte density determines prognosis in colorectal cancer. J. Transl. Med..

[8] Huang P., He P., Tian S., Ma M., Feng P., Xiao H., Mercaldo F., Santone A., Qin J. (2023). A ViT-AMC Network with Adaptive Model Fusion and Multiobjective Optimization for Interpretable Laryngeal Tumor Grading From Histopathological Images. IEEE Trans. Med. Imaging.

[9] Hussain T., Shouno H. (2023). Explainable Deep Learning Approach for Multi-Class Brain Magnetic Resonance Imaging Tumor Classification and Localization Using Gradient-Weighted Class Activation Mapping. Information.

[10] Zeineldin R.A., Karar M.E., Elshaer Z., Coburger J., Wirtz C., Burgert O., Mathis-Ullrich F. (2024). Explainable hybrid vision transformers and convolutional network for multimodal glioma segmentation in brain MRI. Sci. Rep..

[11] Mataraso S.J., Espinosa C.A., Seong D., Reincke S.M., Berson E., Reiss J., Kim Y., Ghanem M., Shu C.H., James T. (2025). A machine learning approach to leveraging electronic health records for enhanced omics analysis. Nat. Mach. Intell..

[12] Aiello M., Esposito G., Pagliari G., Borrelli P., Brancato V., Salvatore M. (2021). How does DICOM support big data management? Investigating its use in medical imaging community. Insights Imaging.

[13] Cirillo D., Valencia A. (2019). Big data analytics for personalized medicine. Curr. Opin. Biotechnol..

[14] Baklola M., Reda Elmahdi R., Ali S., Elshenawy M., Mossad A.M., Al-Bawah N., Mansour R.M. (2025). Artificial intelligence in disease diagnostics: A comprehensive narrative review of current advances, applications, and future challenges in healthcare. Ann. Med. Surg..

[15] Cersosimo A., Zito E., Pierucci N., Matteucci A., La Fazia V.M. (2025). A Talk with ChatGPT: The Role of Artificial Intelligence in Shaping the Future of Cardiology and Electrophysiology. J. Pers. Med..

[16] Msaouel P. (2022). The Big Data Paradox in Clinical Practice. Cancer Investig..

[17] Lu X., Yang C., Liang L., Hu G., Zhong Z., Jiang Z. (2024). Artificial intelligence for optimizing recruitment and retention in clinical trials: A scoping review. J. Am. Med. Inform. Assoc. JAMIA.

[18] Pammi M., Shah P.S., Yang L.K., Hagan J., Aghaeepour N., Neu J. (2025). Digital twins, synthetic patient data, and in-silico trials: Can they empower paediatric clinical trials?. Lancet Digit. Health.

[19] (2024). AI meets real-world patients. Nat. Biotechnol..

[20] Brandenburg J.M., Müller-Stich B.P., Wagner M., van der Schaar M. (2025). Can surgeons trust AI? Perspectives on machine learning in surgery and the importance of eXplainable Artificial Intelligence (XAI). Langenbecks Arch. Surg..

[21] Guha A., Shah V., Nahle T., Singh S., Kunhiraman H.H., Shehnaz F., Nain P., Makram O.M., Mahmoudi M., Al-Kindi S. (2025). Artificial Intelligence Applications in Cardio-Oncology: A Comprehensive Review. Curr. Cardiol. Rep..

[22] Martinez D.S.-L., Noseworthy P.A., Akbilgic O., Herrmann J., Ruddy K.J., Hamid A., Maddula R., Singh A., Davis R., Gunturkun F. (2022). Artificial intelligence opportunities in cardio-oncology: Overview with spotlight on electrocardiography. Am. Heart Hournal Plus Cardiol. Res. Pract..

[23] Krizhevsky A., Sutskever I., Hinton G.E. (2012). ImageNet classification with deep convolutional neural networks. Proceedings of the 26th International Conference on Neural Information Processing Systems.

[24] Carion N., Massa F., Synnaeve G., Usunier N., Kirillov A., Zagoruyko S. (2020). End-to-End Object Detection with Transformers. arXiv.

[25] Ronneberger O., Fischer P., Brox T. (2015). U-Net: Convolutional Networks for Biomedical Image Segmentation. arXiv.

[26] Chen R.J., Lu M.Y., Wang J., Williamson D.F.K., Rodig S., Lindeman N.I., Mahmood F. (2022). Pathomic Fusion: An Integrated Framework for Fusing Histopathology and Genomic Features for Cancer Diagnosis and Prognosis. IEEE Trans. Med. Imaging.

[27] Acosta J.N., Falcone G.J., Rajpurkar P., Topol E.J. (2022). Multimodal biomedical AI. Nat. Med..

[28] Jandoubi B., Akhloufi M.A. (2025). Multimodal Artificial Intelligence in Medical Diagnostics. Information.

[29] Schneider M.A., Linecker M., Fritsch R., Muehlematter U.J., Stocker D., Pestalozzi B., Samaras P., Jetter A., Kron P., Petrowsky H. (2021). Phase Ib dose-escalation study of the hypoxia-modifier Myo-inositol trispyrophosphate in patients with hepatopancreatobiliary tumors. Nat. Commun..

[30] Cancer Simply Explained: What is Cancer and What Can We Do About It?. https://link.springer.com/book/10.1007/978-3-031-84297-9.

[31] Morabito A., De Simone G., Pastorelli R., Brunelli L., Ferrario M. (2025). Algorithms and tools for data-driven omics integration to achieve multilayer biological insights: A narrative review. J. Transl. Med..

[32] Zack M., Stupichev D.N., Moore A.J., Slobodchikov J.D., Sokolov D.G., Trifonov I.F., Gobbs A. (2025). Artificial Intelligence and Multi-Omics in Pharmacogenomics: A New Era of Precision Medicine. Mayo Clin. Proc. Digit. Health.

[33] Drouard G., Mykkänen J., Heiskanen J., Pohjonen J., Ruohonen S., Pahkala K., Lehtimäki T., Wang X., Ollikainen M., Ripatti S. (2024). Exploring machine learning strategies for predicting cardiovascular disease risk factors from multi-omic data. BMC Med. Inf. Decis. Mak..

[34] Wissel D., Rowson D., Boeva V. (2023). Systematic comparison of multi-omics survival models reveals a widespread lack of noise resistance. Cell Rep. Methods.

[35] Muharremi G., Meçani R., Muka T. (2023). The Buzz Surrounding Precision Medicine: The Imperative of Incorporating It into Evidence-Based Medical Practice. J. Pers. Med..

[36] Savage R.S., Ghahramani Z., Griffin J.E., Kirk P., Wild D.L. (2013). Identifying cancer subtypes in glioblastoma by combining genomic, transcriptomic and epigenomic data. arXiv.

[37] Karagoz A. (2025). OmicsCL: Unsupervised Contrastive Learning for Cancer Subtype Discovery and Survival Stratification. arXiv.

[38] Santamarina-Ojeda P., Tejedor J.R., Pérez R.F., López V., Robert A., Mangas C., Fernández A.F., Fraga M.F. (2023). Multi-omic integration of DNA methylation and gene expression data reveals molecular vulnerabilities in glioblastoma. Mol. Oncol..

[39] Aftab M., Mehmood F., Zhang C., Nadeem A., Dong Z., Jiang Y., Liu K. (2025). AI in Oncology: Transforming Cancer Detection through Machine Learning and Deep Learning Applications. arXiv.

[40] Teshale A.B., Htun H.L., Vered M., Owen A.J., Freak-Poli R. (2024). A Systematic Review of Artificial Intelligence Models for Time-to-Event Outcome Applied in Cardiovascular Disease Risk Prediction. J. Med. Syst..

[41] Huang Y., Li J., Li M., Aparasu R.R. (2023). Application of machine learning in predicting survival outcomes involving real-world data: A scoping review. BMC Med. Res. Methodol..

[42] Nikolaou N., Salazar D., RaviPrakash H., Gonçalves M., Mulla R., Burlutskiy N., Markuzon N., Jacob E. (2025). A machine learning approach for multimodal data fusion for survival prediction in cancer patients. npj Precis. Oncol..

[43] Bretthauer M., Wieszczy P., Løberg M., Kaminski M.F., Werner T.F., Helsingen L.M., Mori Y., Holme Ø., Adami H.O., Kalager M. (2023). Estimated Lifetime Gained With Cancer Screening Tests: A Meta-Analysis of Randomized Clinical Trials. JAMA Intern. Med..

[44] Tong D., Tian Y., Zhou T., Ye Q., Li J., Ding K., Li J. (2020). Improving prediction performance of colon cancer prognosis based on the integration of clinical and multi-omics data. BMC Med. Inform. Decis. Mak..

[45] Vonzun L., Brun R., Gadient-Limani N., Schneider M.A., Reding T., Graf R., Limani P., Ochsenbein-Kölble N. (2023). Serum Pancreatic Stone Protein Reference Values in Healthy Pregnant Women: A Prospective Cohort Study. J. Clin. Med..

[46] Cossio M., Wiedemann N., Sanfeliu Torres E., Sole E.B., Igual L. (2025). AI-augmented pathology: The experience of transfer learning and intra-domain data diversity in breast cancer metastasis detection. Front. Oncol..

[47] Orouji S., Liu M.C., Korem T., Peters M.A.K. (2024). Domain adaptation in small-scale and heterogeneous biological datasets. Sci. Adv..

[48] Rapid and Reproducible Multimodal Biological Foundation Model Development with AIDO ModelGenerator. bioRxiv.

[49] Shafi S., Parwani A.V. (2023). Artificial intelligence in diagnostic pathology. Diagn. Pathol..

[50] Komura D., Ochi M., Ishikawa S. (2025). Machine learning methods for histopathological image analysis: Updates in 2024. Comput. Struct. Biotechnol. J..

[51] Niazi M.K.K., Parwani A.V., Gurcan M.N. (2019). Digital pathology and artificial intelligence. Lancet Oncol..

[52] Marble H.D., Huang R., Dudgeon S.N., Lowe A., Herrmann M.D., Blakely S., Leavitt M.O., Isaacs M., Hanna M.G., Sharma A. (2020). A Regulatory Science Initiative to Harmonize and Standardize Digital Pathology and Machine Learning Processes to Speed up Clinical Innovation to Patients. J. Pathol. Inform..

[53] Hassija V., Chamola V., Mahapatra A., Singal A., Goel D., Huang K., Scardapane S., Spinelli I., Mahmud M., Hussain A. (2024). Interpreting Black-Box Models: A Review on Explainable Artificial Intelligence. Cogn. Comput..

[54] Elias P., Jain S.S., Poterucha T., Randazzo M., Lopez Jimenez F., Khera R., Perez M., Ouyang D., Pirruccello J., Salerno M. (2024). Artificial Intelligence for Cardiovascular Care—Part 1: Advances. J. Am. Coll. Cardiol..

[55] Fass L. (2008). Imaging and cancer: A review. Mol. Oncol..

[56] Qureshi I., Yan J., Abbas Q., Shaheed K., Riaz A.B., Wahid A., Khan M.W.J., Szczuko P. (2023). Medical image segmentation using deep semantic-based methods: A review of techniques, applications and emerging trends. Inf. Fusion.

[57] Ranjbarzadeh R., Bagherian Kasgari A., Jafarzadeh Ghoushchi S., Ghoushchi S.J., Anari S., Naseri M., Bendechache M. (2021). Brain tumor segmentation based on deep learning and an attention mechanism using MRI multi-modalities brain images. Sci. Rep..

[58] Punn N.S., Agarwal S. (2022). Modality specific U-Net variants for biomedical image segmentation: A survey. Artif. Intell. Rev..

[59] Rayed M.d.E., Islam S.M.S., Niha S.I., Jim J.R., Kabir M., Mridha M.F. (2024). Deep learning for medical image segmentation: State-of-the-art advancements and challenges. Inform. Med. Unlocked.

[60] He A., Wang K., Li T., Du C., Xia S., Fu H. (2023). H2Former: An Efficient Hierarchical Hybrid Transformer for Medical Image Segmentation. IEEE Trans. Med. Imaging.

[61] Renugadevi M., Narasimhan K., Ramkumar K., Raju N. (2025). A novel hybrid vision UNet architecture for brain tumor segmentation and classification. Sci. Rep..

[62] Neha F., Bhati D., Shukla D.K., Dalvi S.M., Mantzou N., Shubbar S. (2024). U-Net in Medical Image Segmentation: A Review of Its Applications Across Modalities. arXiv.

[63] Kirillov A., Mintun E., Ravi N., Mao H., Rolland C., Gustafson L., Xiao T., Whitehead S., Berg A.C., Lo W.Y. (2023). Segment Anything. arXiv.

[64] Ma J., He Y., Li F., Han L., You C., Wang B. (2024). Segment anything in medical images. Nat. Commun..

[65] Cheng J., Ye J., Deng Z., Chen J., Li T., Wang H., Su Y., Huang Z., Chen J., Jiang L. (2023). SAM-Med2D. arXiv.

[66] Esteva A., Kuprel B., Novoa R.A., Ko J., Swetter S.M., Blau H.M., Thrun S. (2017). Dermatologist-level classification of skin cancer with deep neural networks. Nature.

[67] Chanda T., Hauser K., Hobelsberger S., Bucher T.C., Garcia C.N., Wies C., Kittler H., Tschandl P., Navarrete-Dechent C., Podlipnik S. (2024). Dermatologist-like explainable AI enhances trust and confidence in diagnosing melanoma. Nat. Commun..

[68] Armoundas A.A., Narayan S.M., Arnett D.K., Spector-Bagdady K., Bennett D.A., Celi L.A., Gollob M.H., Hall J.L., Kwitek A.E., Lett E. (2024). Use of Artificial Intelligence in Improving Outcomes in Heart Disease: A Scientific Statement from the American Heart Association. Circulation.

[69] Tomtect. https://shop.tomtect.com/.

[70] Ultromics. https://www.ultromics.com.

[71] Us2.ai. https://us2.ai/.

[72] D’Ascenzo F., Filippo O.D., Gallone G., Mittone G., Deriu M.A., Iannaccone M., Ariza-Solé A., Liebetrau C., Manzano-Fernández S., Quadri G. (2021). Machine learning-based prediction of adverse events following an acute coronary syndrome (PRAISE): A modelling study of pooled datasets. Lancet.

[73] Martínez-Sellés M., Marina-Breysse M. (2023). Current and Future Use of Artificial Intelligence in Electrocardiography. J. Cardiovasc. Dev. Dis..

[74] Ribeiro A.H., Ribeiro M.H., Paixão G.M.M., Oliveira D.M., Gomes P.R., Canazart J.A., Ferreira M.P.S., Andersson C.R., Macfarlane P.W., Meira W. (2020). Automatic diagnosis of the 12-lead ECG using a deep neural network. Nat. Commun..

[75] Hannun A.Y., Rajpurkar P., Haghpanahi M., Tison G.H., Bourn C., Turakhia M.P., Ng A.Y. (2019). Cardiologist-level arrhythmia detection and classification in ambulatory electrocardiograms using a deep neural network. Nat. Med..

[76] Jin Y., Li Z., Wang M., Liu J., Tian Y., Liu Y., Wei X., Zhao L., Liu C. (2024). Cardiologist-level interpretable knowledge-fused deep neural network for automatic arrhythmia diagnosis. Commun. Med..

[77] Bock C., Walter J.E., Rieck B., Strebel I., Rumora K., Schaefer I., Zellweger M.J., Borgwardt K., Müller C. (2024). Enhancing the diagnosis of functionally relevant coronary artery disease with machine learning. Nat. Commun..

[78] Berisha V., Krantsevich C., Hahn P.R., Hahn S., Dasarathy G., Turaga P., Liss J. (2021). Digital medicine and the curse of dimensionality. npj Digit. Med..

[79] Volleberg R.H.J.A., van der Waerden R.G.A., Luttikholt T.J., van der Zande J.L., Cancian P., Gu X., Mol J.-Q., Quax S., Prokop M., Sánchez C.I. (2025). Comprehensive full-vessel segmentation and volumetric plaque quantification for intracoronary optical coherence tomography using deep learning. Eur. Heart J.-Digit. Health.

[80] Lee J., Kim J.N., Gharaibeh Y., Zimin V.N., Dallan L.A.P., Pereira G.T.R., Vergara-Martel A., Kolluru C., Hoori A., Bezerra H.G. (2023). OCTOPUS—Optical coherence tomography plaque and stent analysis software. Heliyon.

[81] Deisenhofer I., Albenque J.-P., Busch S., Gitenay E., Mountantonakis S.E., Roux A., Horvilleur J., Bakouboula B., Oza S., Abbey S. (2025). Artificial intelligence for individualized treatment of persistent atrial fibrillation: A randomized controlled trial. Nat. Med..

[82] Antoun I., Abdelrazik A., Eldesouky M., Li X., Layton G.R., Zakkar M., Somani R., Ng G.A. (2025). Artificial intelligence in atrial fibrillation: Emerging applications, research directions and ethical considerations. Front. Cardiovasc. Med..

[83] Berisha V., Liss J.M. (2024). Responsible development of clinical speech AI: Bridging the gap between clinical research and technology. npj Digit. Med..

[84] Kappen M., Vanhollebeke G., Van Der Donckt J., Van Hoecke S., Vanderhasselt M.-A. (2024). Acoustic and prosodic speech features reflect physiological stress but not isolated negative affect: A multi-paradigm study on psychosocial stressors. Sci. Rep..

[85] Maor E., Perry D., Mevorach D., Taiblum N., Luz Y., Mazin I., Lerman A., Koren G., Shalev V. (2020). Vocal Biomarker Is Associated With Hospitalization and Mortality Among Heart Failure Patients. J. Am. Heart Assoc..

[86] Murton O.M., Dec G.W., Hillman R.E., Majmudar M.D., Steiner J., Guttag J.V., Mehta D.D. (2023). Acoustic Voice and Speech Biomarkers of Treatment Status during Hospitalization for Acute Decompensated Heart Failure. Appl. Sci..

[87] Okada K., Mizuguchi D., Omiya Y., Endo K., Kobayashi Y., Iwahashi N., Kosuge M., Ebina T., Tamura K., Sugano T. (2024). Clinical Utility of Machine Learning-Derived Vocal Biomarkers in the Management of Heart Failure. Circ. Rep..

[88] Kerwagen F., Bauser M., Baur M., Kraus F., Morbach C., Pryss R., Rak K., Frantz S., Weber M., Hoxha J. (2025). Vocal biomarkers in heart failure—Design, rationale and baseline characteristics of the AHF-Voice study. Front. Digit. Health.

[89] Amir O., Abraham W.T., Azzam Z.S., Berger G., Anker S.D., Pinney S.P., Burkhoff D., Shallom I.D., Lotan C., Edelman E.R. (2022). Remote Speech Analysis in the Evaluation of Hospitalized Patients with Acute Decompensated Heart Failure. JACC Heart Fail..

[90] Wanigatunga A., Lu Y., Marino F., Davoudi A., Dougherty R., Schrack J. (2023). Muscle Strength And Incident Dementia in The National Health And Aging Trends Study (NHATS). Innov. Aging.

[91] Xiong P., Lee S.M.-Y., Chan G. (2022). Deep Learning for Detecting and Locating Myocardial Infarction by Electrocardiogram: A Literature Review. Front. Cardiovasc. Med..

[92] Oikonomou E.K., Sangha V., Dhingra L.S., Aminorroaya A., Coppi A., Krumholz H.M., Baldassarre L.A., Khera R. (2025). Artificial Intelligence-Enhanced Risk Stratification of Cancer Therapeutics-Related Cardiac Dysfunction Using Electrocardiographic Images. Circ. Cardiovasc. Qual. Outcomes.

[93] Feeny A.K., Chung M.K., Madabhushi A., Attia Z.I., Cikes M., Firouznia M., Friedman P.A., Kalscheur M.M., Kapa S., Narayan S.M. (2020). Artificial Intelligence and Machine Learning in Arrhythmias and Cardiac Electrophysiology. Circ. Arrhythm. Electrophysiol..

[94] Shil S., Kumar P., Mumbrekar K.D. (2025). Cancer therapy-induced cardiotoxicity: Mechanisms and mitigations. Heart Fail. Rev..

[95] Alshraideh A., Al Fayoumi B., Alshraideh B.M., Alshraideh M. (2025). Hybrid AI Framework for the Early Detection of Heart Failure: Integrating Traditional Machine Learning and Generative Language Models with Clinical Data. Cureus.

[96] Yagi R., Goto S., Katsumata Y., MacRae C.A., Deo R.C. (2022). Importance of external validation and subgroup analysis of artificial intelligence in the detection of low ejection fraction from electrocardiograms. Eur. Heart J. Digit. Health.

[97] Karabayir I., Akbilgic O. (2025). Generalizability of electrocardiographic artificial intelligence. npj Cardiovasc. Health.

[98] Kumar G., Chaudhary P., Quinn A., Su D. (2022). Barriers for cancer clinical trial enrollment: A qualitative study of the perspectives of healthcare providers. Contemp. Clin. Trials Commun..

[99] Mahadik S., Sen P., Shah E.J. (2025). Harnessing digital health technologies and real-world evidence to enhance clinical research and patient outcomes. Digit. Health.

[100] Maru S., Matthias M.D., Kuwatsuru R., Simpson R.J. (2024). Studies of Artificial Intelligence/Machine Learning Registered on ClinicalTrials.gov: Cross-Sectional Study with Temporal Trends, 2010–2023. J. Med. Internet Res..

[101] Yao X., Rushlow D.R., Inselman J.W., McCoy R.G., Thacher T.D., Behnken E.M., Bernard M.E., Rosas S.L., Akfaly A., Misra A. (2021). Artificial intelligence-enabled electrocardiograms for identification of patients with low ejection fraction: A pragmatic, randomized clinical trial. Nat. Med..

[102] Rushlow D.R., Croghan I.T., Inselman J.W., Thacher T.D., Friedman P.A., Yao X., Pellikka P.A., Lopez-Jimenez F., Bernard M.E., Barry B.A. (2022). Clinician Adoption of an Artificial Intelligence Algorithm to Detect Left Ventricular Systolic Dysfunction in Primary Care. Mayo Clin. Proc..

[103] Hollmann N., Müller S., Purucker L., Krishnakumar A., Körfer M., Hoo S.B., Schirrmeister R.T., Hutter F. (2025). Accurate predictions on small data with a tabular foundation model. Nature.

[104] Grossmann R., Schneider M.A., Linecker M., Lehn J.-M., Nicolau C., Traber M., Tay F., Vicente D., Jetter A., Mollet A. (2020). Interprofessional and interdisciplinary collaboration for early phase oncological clinical trials in academia-Myo-inositoltrispyrophophate as model. Pharmacol. Res..

[105] Mazor T., Farhat K.S., Trukhanov P., Lindsay J., Galvin M., Mallaber E., Paul M.A., Hassett M.J., Schrag D., Cerami E. (2025). Clinical Trial Notifications Triggered by Artificial Intelligence–Detected Cancer Progression. JAMA Netw. Open.

[106] Ntinopoulos V., Rodriguez Cetina Biefer H., Tudorache I., Papadopoulos N., Odavic D., Risteski P., Haeussler A., Dzemali O. (2025). Large language models for data extraction from unstructured and semi-structured electronic health records: A multiple model performance evaluation. BMJ Health Care Inform..

[107] Vidovszky A.A., Fisher C.K., Loukianov A.D., Smith A.M., Tramel E.W., Walsh J.R., Ross J.L. (2024). Increasing acceptance of AI-generated digital twins through clinical trial applications. Clin. Transl. Sci..

[108] Böttcher L., Fonseca L.L., Laubenbacher R.C. (2025). Control of medical digital twins with artificial neural networks. Philos. Trans. A Math. Phys. Eng. Sci..

[109] Masison J., Beezley J., Mei Y., Ribeiro H., Knapp A.C., Sordo Vieira L., Adhikari B., Scindia Y., Grauer M., Helba B. (2021). A modular computational framework for medical digital twins. Proc. Natl. Acad. Sci. USA.

[110] Sun T., He X., Li Z. (2023). Digital twin in healthcare: Recent updates and challenges. Digit. Health.

[111] MacKay E.J., Goldfinger S., Chan T.J., Grasfield R.H., Eswar V.J., Li K., Cao Q., Pouch A.M. (2025). Automated structured data extraction from intraoperative echocardiography reports using large language models. Br. J. Anaesth..

[112] Grothey B., Odenkirchen J., Brkic A., Schömig-Markiefka B., Quaas A., Büttner R., Tolkach Y. (2025). Comprehensive testing of large language models for extraction of structured data in pathology. Commun. Med..

[113] Biondi-Zoccai G., D’Ascenzo F., Giordano S., Mirzoyev U., Erol Ç., Cenciarelli S., Leone P., Versaci F. (2025). Artificial Intelligence in Cardiology: General Perspectives and Focus on Interventional Cardiology. Anatol. J. Cardiol..

[114] Eisemann N., Bunk S., Mukama T., Baltus H., Elsner S.A., Gomille T., Hecht G., Heywang-Köbrunner S., Rathmann R., Siegmann-Luz K. (2025). Nationwide real-world implementation of AI for cancer detection in population-based mammography screening. Nat. Med..

[115] Poterucha T.J., Jing L., Ricart R.P., Adjei-Mosi M., Finer J., Hartzel D., Kelsey C., Long A., Rocha D., Ruhl J.A. (2025). Detecting structural heart disease from electrocardiograms using AI. Nature.

[116] Mahayni A.A., Attia Z.I., Medina-Inojosa J.R., Elsisy M.F.A., Noseworthy P.A., Lopez-Jimenez F., Kapa S., Asirvatham S.J., Friedman P.A., Crestenallo J.A. (2021). Electrocardiography-Based Artificial Intelligence Algorithm Aids in Prediction of Long-term Mortality After Cardiac Surgery. Mayo Clin. Proc..

[117] Zondag A.G.M., Rozestraten R., Grimmelikhuijsen S.G., Jongsma K.R., van Solinge W.W., Bots M.L., Vernooij R.W.M., Haitjema S. (2024). The Effect of Artificial Intelligence on Patient-Physician Trust: Cross-Sectional Vignette Study. J. Med. Internet Res..

[118] Akingbola A., Adeleke O., Idris A., Adewole O., Adegbesan A. (2024). Artificial Intelligence and the Dehumanization of Patient Care. J. Med. Surg. Public Health.

[119] Nong P., Ji M. (2025). Expectations of healthcare AI and the role of trust: Understanding patient views on how AI will impact cost, access, and patient-provider relationships. J. Am. Med. Inform. Assoc. JAMIA.

[120] Sagona M., Dai T., Macis M., Darden M. (2025). Trust in AI-assisted health systems and AI’s trust in humans. npj Health Syst..

[121] Maleki Varnosfaderani S., Forouzanfar M. (2024). The Role of AI in Hospitals and Clinics: Transforming Healthcare in the 21st Century. Bioengineering.

[122] Muka T., Glisic M., Milic J., Verhoog S., Bohlius J., Bramer W., Chowdhury R., Franco O.H. (2020). A 24-step guide on how to design, conduct, and successfully publish a systematic review and meta-analysis in medical research. Eur. J. Epidemiol..

[123] Foote H.P., Hong C., Anwar M., Borentain M., Bugin K., Dreyer N., Fessel J., Goyal N., Hanger M., Hernandez A.F. (2025). Embracing Generative Artificial Intelligence in Clinical Research and Beyond: Opportunities, Challenges, and Solutions. JACC Adv..

[124] Marey A., Arjmand P., Alerab A.D.S., Eslami M.J., Saad A.M., Sanchez N., Umair M. (2024). Explainability, transparency and black box challenges of AI in radiology: Impact on patient care in cardiovascular radiology. Egypt. J. Radiol. Nucl. Med..

[125] Lou Y., Caruana R., Gehrke J. Intelligible models for classification and regression. Proceedings of the 18th ACM SIGKDD International Conference on Knowledge Discovery and Data Mining.

[126] Javed H., El-Sappagh S., Abuhmed T. (2024). Robustness in deep learning models for medical diagnostics: Security and adversarial challenges towards robust AI applications. Artif. Intell. Rev..

[127] Balendran A., Beji C., Bouvier F., Khalifa O., Evgeniou T., Ravaud P., Porcher R. (2025). A scoping review of robustness concepts for machine learning in healthcare. npj Digit. Med..

[128] Sharkey L., Chughtai B., Batson J., Lindsey J., Wu J., Bushnaq L., Goldowsky-Dill N., Heimersheim S., Ortega A., Bloom J. (2025). Open Problems in Mechanistic Interpretability. arXiv.

[129] Schömig-Markiefka B., Pryalukhin A., Hulla W., Bychkov A., Fukuoka J., Madabhushi A., Achter V., Nieroda L., Büttner R., Quaas A. (2021). Quality control stress test for deep learning-based diagnostic model in digital pathology. Mod. Pathol..

[130] Mittelstadt B. (2019). Principles alone cannot guarantee ethical AI. Nat. Mach. Intell..

[131] Shen J., Zhang C.J.P., Jiang B., Chen J., Song J., Liu Z., He Z., Wong S.Y., Fang P.-H., Ming W.-K. (2019). Artificial Intelligence Versus Clinicians in Disease Diagnosis: Systematic Review. JMIR Med. Inform..

[132] Parikh R.B., Obermeyer Z., Navathe A.S. (2019). Regulation of predictive analytics in medicine. Science.

[133] Char D.S., Shah N.H., Magnus D. (2018). Implementing Machine Learning in Health Care—Addressing Ethical Challenges. N. Engl. J. Med..

[134] Mehrabi N., Morstatter F., Saxena N., Lerman K., Galstyan A. (2021). A Survey on Bias and Fairness in Machine Learning. ACM Comput. Surv..

[135] Zech J.R., Badgeley M.A., Liu M., Costa A.B., Titano J.J., Oermann E.K. (2018). Variable generalization performance of a deep learning model to detect pneumonia in chest radiographs: A cross-sectional study. PLoS Med..

[136] Reddy S., Allan S., Coghlan S., Cooper P. (2020). A governance model for the application of AI in health care. J. Am. Med. Inform. Assoc. JAMIA.

[137] Celi L.A., Cellini J., Charpignon M.-L., Dee E.C., Dernoncourt F., Eber R., Mitchell W.G., Moukheiber L., Schirmer J., Situ J. (2022). Sources of bias in artificial intelligence that perpetuate healthcare disparities—A global review. PLoS Digit. Health.

[138] Paik K.E., Hicklen R., Kaggwa F., Puyat C.V., Nakayama L.F., Ong B.A., Shropshire J.N.I., Villanueva C. (2023). Digital Determinants of Health: Health data poverty amplifies existing health disparities—A scoping review. PLoS Digit. Health.

[139] Benjamens S., Dhunnoo P., Meskó B. (2020). The state of artificial intelligence-based FDA-approved medical devices and algorithms: An online database. npj Digit. Med..

[140] Topol E.J. (2019). High-performance medicine: The convergence of human and artificial intelligence. Nat. Med..

[141] Kelly C.J., Karthikesalingam A., Suleyman M., Corrado G., King D. (2019). Key challenges for delivering clinical impact with artificial intelligence. BMC Med..

[142] He J., Baxter S.L., Xu J., Xu J., Zhou X., Zhang K. (2019). The practical implementation of artificial intelligence technologies in medicine. Nat. Med..

[143] Jiang F., Jiang Y., Zhi H., Dong Y., Li H., Ma S., Wang Y., Dong Q., Shen H., Wang Y. (2017). Artificial intelligence in healthcare: Past, present and future. Stroke Vasc. Neurol..

[144] Wang F., Preininger A. (2019). AI in Health: State of the Art, Challenges, and Future Directions. Yearb. Med. Inform..

[145] Ilcheva L., Risteski P., Tudorache I., Häussler A., Papadopoulos N., Odavic D., Rodriguez Cetina Biefer H., Dzemali O. (2023). Beyond Conventional Operations: Embracing the Era of Contemporary Minimally Invasive Cardiac Surgery. J. Clin. Med..

[146] Davenport T., Kalakota R. (2019). The potential for artificial intelligence in healthcare. Future Healthc. J..

[147] Hagendorff T. (2020). The Ethics of AI Ethics: An Evaluation of Guidelines. Minds Mach..

[148] Guo C., Pleiss G., Sun Y., Weinberger K.Q. (2017). On Calibration of Modern Neural Networks. arXiv.

[149] Ovadia Y., Fertig E., Ren J., Nado Z., Sculley D., Nowozin S., Dillon J.V., Lakshminarayanan B., Snoek J. (2019). Can You Trust Your Model’s Uncertainty? Evaluating Predictive Uncertainty Under Dataset Shift. arXiv.

[150] Gal Y., Ghahramani Z. (2016). Dropout as a Bayesian Approximation: Representing Model Uncertainty in Deep Learning. arXiv.

[151] Lakshminarayanan B., Pritzel A., Blundell C. (2017). Simple and Scalable Predictive Uncertainty Estimation using Deep Ensembles. Advances in Neural Information Processing Systems.

[152] Zaidi S., Zela A., Elsken T., Holmes C., Hutter F., Teh Y.W. (2022). Neural Ensemble Search for Uncertainty Estimation and Dataset Shift. arXiv.

[153] Jain S., Agrawal A., Saporta A., Truong S.Q.H., Duong D.N., Bui T., Chambon P., Zhang Y., Lungren M.P., Ng A.Y. (2021). RadGraph: Extracting Clinical Entities and Relations from Radiology Reports. arXiv.

[154] Min S., Krishna K., Lyu X., Lewis M., Yih W.-T., Koh P.W., Iyyer M., Zettlemoyer L., Hajishirzi H. (2023). FActScore: Fine-grained Atomic Evaluation of Factual Precision in Long Form Text Generation. arXiv.

[155] Moor M., Banerjee O., Abad Z.S.H., Krumholz H.M., Leskovec J., Topol E.J., Rajpurkar P. (2023). Foundation models for generalist medical artificial intelligence. Nature.

[156] Tu T., Azizi S., Driess D., Schaekermann M., Amin M., Chang P.-C., Carroll A., Lau C., Tanno R., Ktena I. (2023). Towards Generalist Biomedical AI. arXiv.

[157] Liu X., Faes L., Kale A.U., Wagner S.K., Fu D.J., Bruynseels A., Mahendiran T., Moraes G., Shamdas M., Kern C. (2019). A comparison of deep learning performance against health-care professionals in detecting diseases from medical imaging: A systematic review and meta-analysis. Lancet Digit. Health.

[158] Masters K. (2019). Artificial intelligence in medical education. Med. Teach..

[159] Kolachalama V.B., Garg P.S. (2018). Machine learning and medical education. npj Digit. Med..

[160] Mueller A., Siems J., Nori H., Salinas D., Zela A., Caruana R., Hutter F. (2024). GAMformer: In-Context Learning for Generalized Additive Models. arXiv.

